# Polypyrrole/tannin biobased nanocomposite with enhanced electrochemical and physical properties

**DOI:** 10.1039/c7ra13378b

**Published:** 2018-01-15

**Authors:** Mahnaz M. Abdi, Nur Farhana Waheeda Mohd Azli, Hong Ngee Lim, Paridah Md Tahir, Gholamreza Karimi, Yeoh Beng Hoong, Mohammad Khorram

**Affiliations:** Department of Chemical Engineering, Shiraz University Shiraz 7134851154 Iran mahnaz@upm.edu.my mahnazm.abdi@shirazu.ac.ir; Department of Chemistry, Faculty of Science, Universiti Putra Malaysia 43400 UPM Serdang Selangor Malaysia; Institute of Tropical Forestry and Forest Products, Universiti Putra Malaysia 43400 UPM Serdang Selangor Malaysia; Fiber Development Centre, Malaysian Timber Industry Board Olak Lempit, Banting Selangor Malaysia

## Abstract

In this research, tannin (TA) extracted from *Acacia mangium* and a cationic surfactant, cetyltrimethylammonium bromide (CTAB), were used to modify and enhance the physical and electrochemical properties of a polypyrrole (PPy) composite. Brunauer–Emmett–Teller (BET) analysis presented a higher degree of surface area and porosity for the PPy/TA/CTAB nanocomposite. A highly porous and rod like structure with a lumpy surface was observed for PPy/TA prepared in the presence of CTAB by Field Emission Scanning Electron Microscopy (FESEM) and Transmission Electron Microscopy (TEM). Cyclic voltammograms of the modified SPE electrode using PPy/TA/CTAB displayed an enhanced current response compared to the electrode modified with only PPy or PPy/TA. Electrochemical Impedance Spectroscopy (EIS) exhibited a lower value of charge transfer resistance (*R*_ct_) and higher electron transfer for the modified electrode, making the nanocomposite a promising candidate for biosensor application.

## Abstract 2

Tannin (TA) extracted from *Acacia mangium* and a cationic surfactant, cetyltrimethylammonium bromide (CTAB), were used to modify and enhance the physical and electrochemical properties of polypyrrole (PPy) composite.

## Graphical abstract

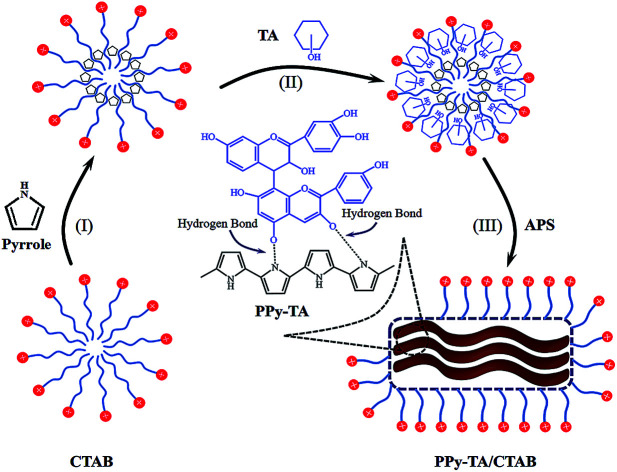

## 1. Introduction

Tannins (TAs) are a group of phenolic compounds containing sufficient hydroxyls and other suitable groups such as carboxyls, to give them the ability to form strong complexes with various macromolecules. Based on the specific structural characteristics and chemical properties of TAs, they can be divided into the flavonoid-derived condensed tannins, and hydrolysable tannins.^1,2^ One of the main features of condensed tannins is their affinity to bind and precipitate proteins which are affected by the type, molecular mass, and structure of the tannins,^3^ and the quality of the fiber.^4^ The condensed tannin from *Acacia mangium* is a type of natural phenolic substance which comes from the repeating unit of condensed tannins called profisetinidins. The high levels of natural phenols (catechin) in the extractible content of *Acacia* bark make it a good potential alternative for synthetic phenols.^5^ Due to the content of flavonoid and phenolic compounds, TAs are widely used in a variety of applications such as the food industry,^6,7^ natural healing for medical and pharmaceutical uses,^8–11^ adhesive resins,^12^ and corrosion inhibition,^13^ while only a few studies have been reported on the application of tannins in sensors.^14,15^

In this research condensed tannin with sufficient hydroxyls was introduced into polypyrrole to prepare a composite of PPy/TA with enhanced mechanical and electrochemical properties of the composites. The composite of PPy and TA synergistically combine the electrical properties of conducting polymers (CPs) with the structural advantages of bio-based polymers that is useful in different applications. Polypyrrole (PPy), one of the well-known conducting polymers, has attracted special interest due to its high conductivity, ease and high flexibility in preparation, biocompatibility, environmental stability, and its electrochemical properties. However, despite all its advantages, PPy lacks mechanical properties and has a low level of solubility and dispersibility in common organic solvents that making it difficult to be coated on electrodes by conventional coating methods which limits its extensive applications.^16^ In order to improve the structural and physical properties of this material, several attempts have been made to modify PPy including blends or composite materials using biopolymers, nanoparticles and surfactants.^17–19^

The aim of this research was to develop a nanocomposite of CPs by the chemical polymerization of pyrrole in the presence of tannin (TA), and a cationic surfactant, cetyltrimethylammonium bromide (CTAB). Tannin with high bond strength and good mechanical properties is able to enhance the physical and structural properties of the resultant polymer. It has also been shown that TA serves as a dopant during polymerization and due to its large molecular size, it is able to be deeply entrapped in the PPy backbone which later repels unwanted biomolecules such as ascorbic acid in selective biosensor application.^14^ However, the bulky structure and low surface area of TA composite limits its application in electrode modification. Thus, the use of a surfactant will counter this limitation.

Surfactants are one of the materials that have been used to modify CPs, leading to many applications in sensing devices. In the current research, a cationic surfactant (CTAB) was used as a soft template to prepare a nanostructured compound for developing a high porous substrate with high electron transferring properties. The composites of PPy–TA were prepared in the presence of the best selected composition of CMC for CTAB and being used for other characterization. The critical micelle concentration (CMC) is the concentration of surfactant above which the micelles form and after that surface tension remains relatively constant.

## 2. Materials and methods

### 2.1 Materials

Pyrrole, ammonium persulphate (APS) and CTAB were provided by Sigma Aldrich Chemie GmbH. The pyrrole monomer was purified by distillation before use. Condensed tannin was provided by the Institute of Tropical Forestry and Forest Products, Universiti Putra Malaysia (INTROP) as described in following part. The rest of the chemicals were of the highest analytical grade and used without further purification. The solutions were prepared using de-ionized water (DIW) from a Mili-Q ultrapure water system with a resistivity of 18 MΩ cm.

### 2.2 Preparation of the composites

In this research, *Acacia mangium* was obtained from the processing mills in Malaysia located at Mentakab, Lembah Beringin, Telaga and Tawau. Condensed tannins (TA) were prepared in the Institute of Tropical Forestry and Forest Products (INTROP), UPM. The collected bark extracts of *Acacia mangium* were prepared as described by Hoong *et al.*^20^ The bark was chipped and ground into fine particles with a size less than 1 mm. The fine bark was further extracted with hot water at 75 °C in a water bath for 3 hours. The mixture was first screened through a fine filter (140 mesh) and then filtered on a sintered glass followed by concentrated to a 40–50% solids under a reduced pressure at 50–55 °C and lastly dried in an oven at 50 °C until the weight was constant. The solution of pyrrole and CTAB were prepared by adding different concentration of CTAB ranging from 2 to 12 cmc to 0.1 M of pyrrole in DIW. The cmc of CTAB is 0.87 mM.^21^ The tannin solutions were prepared separately by dissolving different concentrations of tannin powder ranging from 0.003–0.02 M in 10–20 ml DIW until TA was homogenized completely. Both Py–CTAB and tannin solutions were kept in an ice bath until the temperature reached between 1 to 5 °C. Then, both prepared solutions were mixed together by stirring for 5–10 minutes while kept in an ice bath. Later, the subsequent solution was oxidized by a dropwise addition of APS while stirring in an ice bath for 30 minutes to obtain a black and homogenous precipitate. The mixture was left at room temperature for 2 hours and then centrifuged and washed with DIW until the supernatant was clear. The precipitate, PPy–TA–CTAB, was then transferred onto a watch glass and dried at 60 °C for 24 hours, after which the resulting black powder was collected. The PPy–TA composite was prepared using the same procedure without adding CTAB.

### 2.3 Fabrication of the modified electrode

1 mg of the chemically prepared composite was dispersed in 2 ml deionized water and ultra-sonicated for 15 minutes at rated frequency of 50/60 Hz to obtain a stable suspension. To obtain a homogeneous and uniform surface of the sample on the electrode, 10 μl of suspension was dropped onto the screen printed electrode (SPE), in 2–3 steps of drop casting followed by drying at room temperature.

### 2.4 Characterization techniques

The surface morphology of the nanocomposites was analyzed using a field emission scanning electron microscope (FESEM, JEOL JSM-7600F). Surface area determination was done by Brunauer–Emmett–Teller analysis (BET, 3Flex Version 1.02, Micrometrics). The nitrogen adsorption–desorption isotherms were obtained by using a high performance 3Flex instrument where nitrogen gas is generally employed as the probe molecule and is exposed to solid under investigation at liquid nitrogen conditions (77 K). Thermal stability measurement was done by Thermogravimetric Analysis (TGA/SDTA 851, Mettler Toledo). Transmission Electron Microscopy (TEM) was done using a JEOL JEM 2100 Field Emission TEM and Fourier transform infrared spectroscopy (FTIR, Perkin Elmer Spectrum) was used to study the chemical structure of the composites in the range of 4000–400 cm^−1^. A potentiostat Autolab 204 from Metrohm was used for all the electrochemical measurements with a Nova software version 1.11. The CV measurements were carried out at a potential range from −1.5 V to 1.5 V with a scan rate of 100 mV s^−1^. The EIS measurements were performed with a frequency ranging from 0.1 Hz to 100 kHz at an AC amplitude of 5 mV.

## 3. Results and discussion

**Fig. 1:**
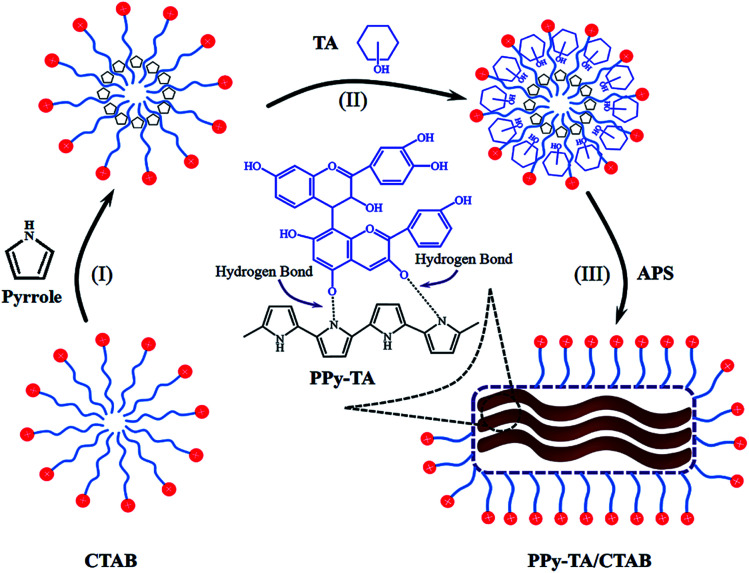
Schematic view of chemical polymerization of pyrrole in the presence of TA and CTAB.

During composite preparation, the hydrophobic pyrrole molecules will locate themselves at the interior of micelles in aqueous solution of the cationic surfactant CTAB. The micelles developed from the self-assembly of surfactant molecules serves as the template during the polymerization process and play an important role in tailoring the nanostructure of PPy.^22^ Tannins have important hydrophobic interactions together with strong hydrogen bounding tendencies through their hydroxyl groups. These hydrophobic nature and tendencies to have hydrogen bonding with pyrrole helps tannins to diffuse inside micelles and form the composite of PPy/TA during oxidative polymerization by APS. Fig. 1 displays schematic view of chemical polymerization of pyrrole/CTAB in the presence of tannin.

### 3.1 Morphology

**Fig. 2:**
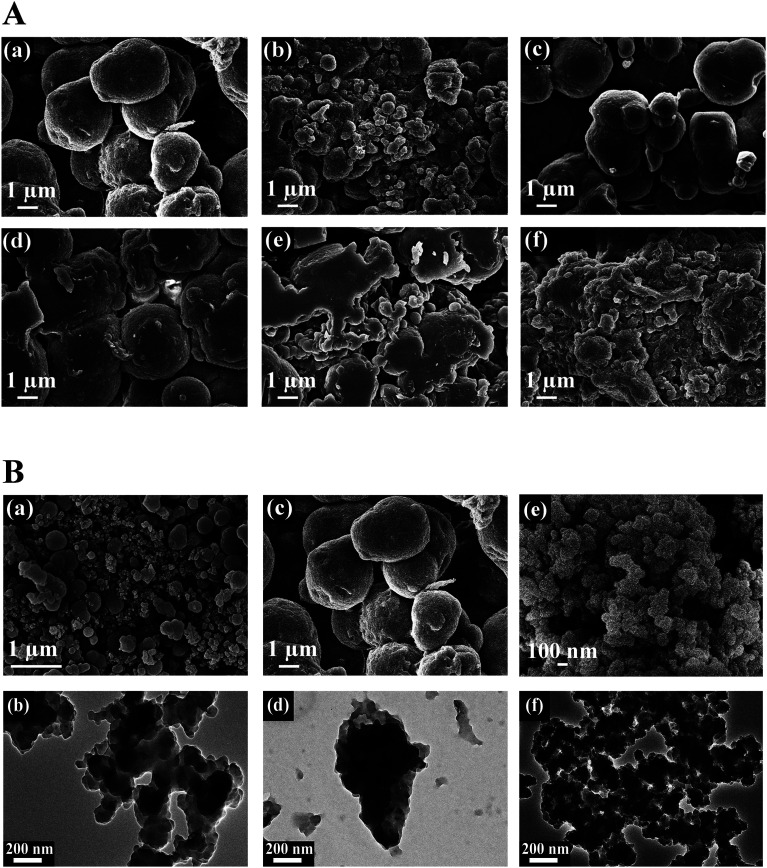
(A) FESEM of PPy/TA microstructures at different concentrations of TA (a) 0.003 M, (b) 0.004 M, (c) 0.005 M, (d) 0.006 M, (e) 0.01 M, and (f) 0.02 M with scale bar of 1 μm. (B) FESEM and TEM images of (a, b) pure PPy (c, d) PPy/TA scale bar 1 μm and (e, f) PPy/TA/CTAB nanocomposites, scale bar 100 nm. TEM for all images are with 200 nm scale bar.

The morphology of the PPy–TA composites prepared from a solution containing 0.02 M of Py and different concentrations of TA is shown in Fig. 2A. The sizes of the spherical particles increased after adding 0.003 M TA to the solution containing pyrrole and by increasing the TA content, some irregular shapes were observed in the composite (Fig. 2A(a–d)). At high concentrations of TA, the globular structure completely disappeared and a shapeless composite mass was obtained (Fig. 2A(e, f)). However, the bulky and the shapeless structure of PPy–TA changed to a rod like structure with a lumpy surface when the composite was prepared in the presence of CTAB. The size of the particles was between 1–3 μm that was aggregated in some parts. It seemed that this lumpy structure increased the porosity of the composite. The differences in the morphologies of PPy, PPy/TA and PPy/TA/CTAB are shown in Fig. 2B. A globular, dense and nonporous structure around 1 μm was observed for PPy prepared from a solution containing 0.2 M pyrrole (Fig. 2B(a, b)). The TEM images of the composites were also compared. The images of PPy/TA (Fig. 2B(c, d)) revealed that TA was able to be entrapped in the backbone of PPy increasing the size of spherical particles, while CTAB was able to tailor the shape of the PPy and made it less agglomerated, and smaller in size (Fig. 2B(e, f)). CTAB was found to have a significant influence on the formation of the nanostructure of the PPy composite which was related to its function as a soft-template for the polymerization of pyrrole by the self-assembly of the cationic surfactant and monomer.^21^

### 3.2 Electrochemical behavior

The electrochemical properties of the modified SPE electrodes were investigated by cyclic voltammetry (CV) in a solution of phosphate buffer saline solution (PBS) at a pH of 7.0. The electrochemical responses were studied in the range of −1.5 V to 1.5 V with a potential scan rate of 0.1 V s^−1^ at room temperature.

**Fig. 3:**
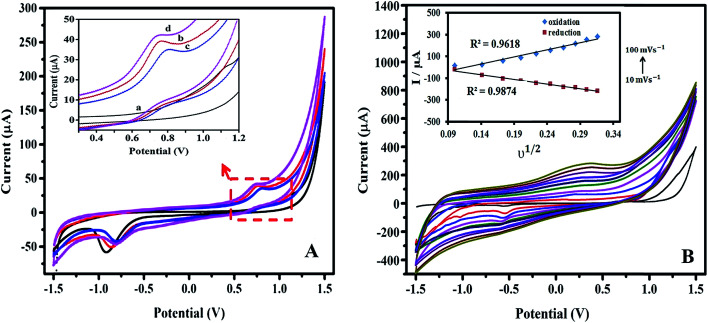
(A) Cyclic voltammograms of (a) bare SPE (b) SPE/PPy, (c) SPE/PPy/TA and (d) SPE/PPy/TA/CTAB. Scan rates: 0.1 V s^−1^. Inset plot shows an enlarged view of the anodic peak. (B) Cyclic voltammograms of PPy/TA/CTAB modified electrode with different scan rate. Inset plot shows oxidation and reduction peaks against square root of scan rate. All was done in PBS buffer solution (pH 7.0).

Fig. 3A shows the cyclic voltammograms of bare SPE, SPE/PPy, SPE/PPy/TA and SPE/PPy/TA/CTAB. To prepare the SPE/PPy/TA modified electrode, the composite was prepared from a solution containing 0.1 M pyrrole and 0.006 M TA and the SPE/PPy/TA/CTAB modified electrode was fabricated from a solution containing 0.1 M pyrrole, 0.006 M TA and 6 CMC of CTAB. The voltammogram of the bare SPE showed the lowest current response for both the anodic and cathodic peaks while SPE/PPy displayed an enhanced anodic current due to the ion transport properties of PPy doped by APS. The oxidation and reduction peaks appeared at 0.76 V and −0.93 V that were assigned to the redox peaks of the porphyrin ring.^23^ However, PPy has a bulky structure, as proven by FESEM, which caused the relatively slow electron transferring.

The PPy/TA modified electrode presented a lower current response, as confirmed by BET and SEM results. PPy/TA displayed lower surface area and porosity compared to the PPy lowering electron transferring. In addition, the large-sized tannin molecules entrapped in the polymer backbone could not be removed easily from the polymer when compared to smaller dopants.^24^ At a working pH of 7, the phenolic groups ionize and tannin become negatively charged which enables it to take small-sized cations inside the polymer during reduction and eliminate them during the oxidation process.^14^ The cyclic voltammogram of SPE/PPy/TA/CTAB modified electrode revealed the highest anodic current that was possibly due to the enhancement of composite's porosity which allowed faster electron transferring. Therefore, it was proven that the electrochemical properties of the composite were remarkably enhanced compared to the PPy and PPy/TA composites.

In order to further investigate the electrochemical behavior of the modified electrode using the nanocomposites, the effect of scan rate on the current response was studied. The CVs of the PPy/TA/CTAB modified electrode in various scan rates ranging from 10 to 100 mV s^−1^ in buffer solution with a pH of 7.0 are shown in Fig. 3B. The anodic and cathodic currents linearly increased with the square root of scan rate confirming diffusion controlled process on the surface of the electrode. It means that the electron transfer at the working electrode surface is fast and the current is limited by the diffusion of the analyte species to the electrode surface. The inset plot in Fig. 3B shows the corresponding plot of peak currents *vs.* square root of scan rates. The *R*^2^ value (linear regression) of the anodic peak and cathodic peaks were 0.9618 and 0.9874, respectively.

**Fig. 4:**
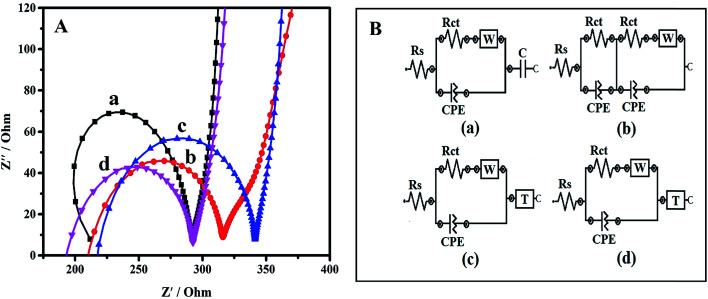
(A) Nyquist plots of (a) bare SPE, (b) PPy, (c) PPy/TA and (d) PPy/TA/CTAB in PBS pH 7. (B) Randles equivalent circuit models of (a) bare SPE, (b) PPy, (c) PPy/TA and (d) PPy/TA/CTAB modified electrodes.

The behavior of all the electrode/electrolyte systems and impedance changes of the electrode surfaces were effectively studied by EIS. The electron transfer characteristics and electron recombination processes at the electrode interfacial surfaces in phosphate buffer solution (pH 7.0) were investigated and are shown as Nyquist plots in Fig. 4A. Impedance measurements are able to provide information on the charge transfer resistance (*R*_ct_) associated with the rate of electron transfer between the redox species and the different electrode surface, which is equal to the diameter of the semicircle. At higher frequencies the semicircle presents the electron transfer process, whereas the linear part at lower frequencies presents the diffusion process. The electrical properties of the electrode surface can be interpreted by fitting the experimental results to the equivalent circuits using the nonlinear least-squares fitting procedure.^25^

The circuit usually consists of the combination of resistances and capacitances. The solution resistance (*R*_s_) represents the solution-phase interference that arises primarily from the electrolyte resistance, while Warburg impedance (*W*) associated to the impedance of diffusive ion transport. Capacitance (*C*) is described as the ability of the circuit to store electric charge. A constant phase element (CPE) labeled with *Q* in the circuit, models the behavior of an imperfect double layer capacitor. CPE was introduced instead of a pure capacitor in the fitting procedure to obtain a good agreement between the simulated and experimental data, where the semicircles in the Nyquist plots are depressed due to surface roughness, heterogeneity of the surface, or other effects that cause uneven current distributions on the electrode surface.^26^ The *T* in the circuit element is called as hyperbolic tangent which useful to describe the diffusion process. Thus, in this study, *R*_ct_ was the main variable and a significant part of impedance, which being used for description of electrode surface behavior.

Fig. 4A shows the Nyquist plot (*Z*′′ *vs. Z*′) for the bare SPE, PPy, PPy/TA and PPy/TA/CTAB and the results have been presented in Table 1. The electrode modified with SPE displayed a large semicircle with an almost straight tail line for bare confirmed the high charge transfer resistance occurring at the surface of the electrode. It was observed that the deposition of the PPy on the bare SPE caused a large decrease in the *R*_ct_ due to the conductive properties of the PPy. With the deposition of the PPy/TA, a slight increment in the *R*_ct_ was observed for PPy/TA modified electrode that could possibly due to the TA content which does not have the ability to conduct current as well as the bulky surface of the modified electrode. However, the value of *R*_ct_ dramatically decreased as the PPy/TA/CTAB was deposited on the SPE represented an enhanced reaction rate kinetics which is ascribed to the formation of nanosized composite with high surface area providing higher charge flow transfer and a higher conductivity of the composites.

**Table 1:** The *R*_ct_ of the bare and modified electrodes

Electrode	*R*_s_ (Ω)	*R*_ct_ (Ω cm^2^)
Bare SPE	178	142
PPy	209	109
PPy/TA	223	119
PPy/TA/CTAB	197	98

Further understanding on the impedance behavior of the bare SPE and modified electrodes are shown by Randles equivalent circuit model (Fig. 4B). The circuits were obtained by fitting the experimental results using nonlinear least-squares fitting procedure. Circuit's elements were specified by symbols like *R* and *C*, in order to translate into an equivalent circuit and easy to specify the circuit obtained. The equivalent circuit of the simple electrochemical cell with *R*_s_, *R*_ct_, and CPE of bare SPE and PPy exhibited by [*R*([*RW*]*Q*)*C*] and [*R*(*RQ*)([*RW*]*Q*)] circuit modelling, respectively (Fig. 4B(a and b)). Both PPy/TA and PPy/TA/CTAB displayed [*R*([*RW*]*Q*)*T*] circuit modelling (Fig. 4B(c and d)). The brackets show that the enclosed elements were parallel and square brackets are used for elements in series. The obtained results are in a good agreement with the results of cyclic voltammetry.

### 3.3 Chemical and physical properties

**Fig. 5:**
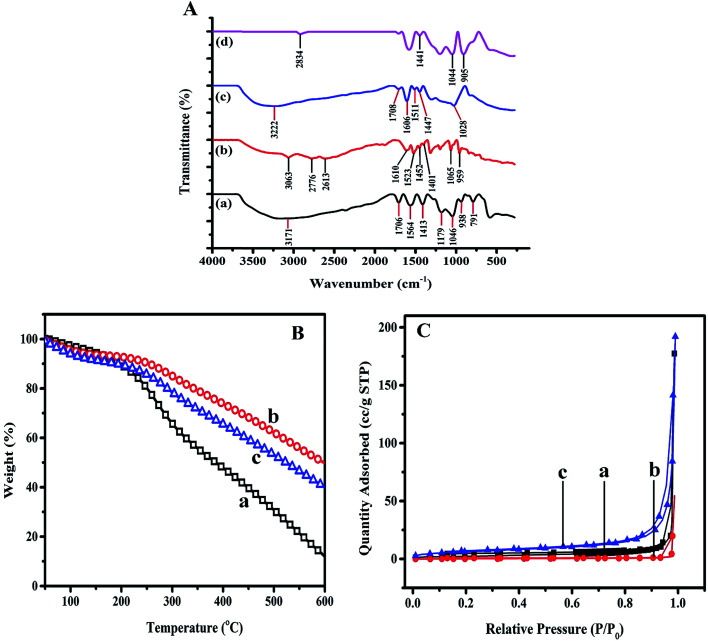
(A) FT-IR spectra of (a) PPy, (b) TA, (c) PPy/TA and (d) PPy/TA/CTAB composites. (B) TGA thermograms of (a) PPy, (b) PPy/TA and (c) PPy/TA/CTAB nanocomposite. (C) Nitrogen adsorption–desorption isotherm of (a) PPy (b) PPy/TA and (c) PPy/TA/CTAB at −196 °C in relative pressure range from 0.009 to 0.8.

The infrared spectra of the PPy, TA, PPy/TA and PPy/TA/CTAB nanocomposites were recorded in a range of 280–4000 cm^−1^ and the FTIR spectra were presented in Fig. 5A. The broad band in the range of 2000–4000 cm^−1^, which is known as the “tail of the electronic absorption band” is the characteristics of the conducting polymers that confirmed PPy preparation. A broad band at 3171 cm^−1^ was observed for PPy that was corresponding to the N–H and C–H stretching vibrations,^27^ while the peak at 1706 cm^−1^ corresponding to the C=C symmetrical stretching. The peaks observed at 1564 cm^−1^ and 1413 cm^−1^ related to the ring C=C stretching and pyrrole C–N stretching, respectively. The peak at 1179 cm^−1^ was assigned to the C–H in plane deformation while the peak at 1046 cm^−1^ corresponded to N–H in plane bending deformation.^28,29^ The peak at 938 cm^−1^ and 791 cm^−1^ assigned to the =C–H out-of-plane vibrations.^30^ Presence all these characteristic absorption peaks confirmed the formation of PPy doped by APS.

The infrared spectrum of TA represented the broad peaks in the region of 3500–3000 cm^−1^ were assigned to O–H stretching vibration.^31^ The peaks at 3063 cm^−1^ and 2776 cm^−1^ related to the =C–H vibration and asymmetric stretching of C–H groups for methylene substituents which was in agreement with that reported in literature.^32^

The peaks at 1610 cm^−1^ and 1523 cm^−1^ were attributed to the aromatic ring stretching vibrations that shifted to the 1606 cm^−1^ and 1511 cm^−1^ in PPy–TA composite indicating the incorporation of TA inside the PPy structure, The peaks observed at 1452 cm^−1^ and 1401 cm^−1^ represented C=C aromatic frame stretching and O–H in plane deformation.^33^ The C–O stretching vibration and aromatic C–H out of plane bending vibration in TA were observed at 1065 cm^−1^ and 959 cm^−1^, respectively.^32^ The peak observed at 1452 cm^−1^ was assigned to the C=C aromatic frame stretching that shifted to 1447 cm^−1^ in the PPy/TA composite confirming the presence of TA in the composite structure. The spectrum of the PPy/TA composite showed a broad absorbance at approximately 3222 cm^−1^ that could be possibly due to the overlapping of the C–H stretching of PPy and the hydroxyl group of TA. Another important peak related to the N–H in-plane deformation of pyrrole ring was observed at 1028 cm^−1^ in the PPy/TA composite confirming the formation of the PPy composite with TA. The presence of CTAB was detected at 905 cm^−1^ in the PPy/TA/CTAB composite that corresponded to the alkane C–C of CTAB. The peaks related to the O–H bending of TA and N–H stretching of PPy were observed at 1441 cm^−1^ and 1044 cm^−1^ in the PPy/TA/CTAB spectrum, respectively (Fig. 5A(d)).

Fig. 5B displays the thermal degradation profiles for PPy, PPy/TA and PPy/TA/CTAB nanocomposite. The TGA weight loss curve of all samples was plotted from 50 °C to 600 °C at a scan rate of 10 °C min^−1^ under nitrogen gas (N_2_). The first weight loss contributed to the desorption of physisorbed water for PPy occurred in the range of 50–100 °C and weight loss was 4.04%. The second stage in the range of 123–320 °C attributed to the degradation of counterion, persulphate while the major weight loss of 83.74% related to polymer backbone degradation of PPy occurred in the range of 320–600 °C. About 12.24% of weight percentage remained around 600 °C which are in the agreement with literature review.^34^ Thermal analysis of the PPy/TA composite has also been shown in Fig. 5B. The first weight loss is described as the removal of adsorbed water and the second loss ascribed to the dopant ion. The final degradation of polymer was due to the composite backbone degradation that occurred above the temperature of 300 °C. Generally, the decomposition of TA takes place at the temperature of 210 °C and it burned at the temperature of 526 °C.^35^ Based from TGA obtained for PPy/TA, the maximum decomposition temperature of the PPy/TA composite increased to 307 °C (compared to PPy) which indicates the presence and interaction of TA with PPy chain *via* hydrogen bonding and hydrophobic interactions.^15^

All results showed the thermal stability enhancement of PPy in the presence of TA. However, adding CTAB to the composite lead to the decreasing in the thermal stability of the nancomposite. CTAB transformations against the temperature during calcination of CTAB up to ∼250 °C is well known.^36^ CTAB completely could be decomposed where there was no trace of CTAB at the temperature from 200–500 °C.^37^ (TGA of CTAB has not shown here).

The TGA obtained for PPy/TA/CTAB nanocomposite presented the maximum decomposition of the composite at the temperature of 295 °C, lower than that for PPy/TA. The residue remained at 600 °C for PPy/TA/CTAB nanocomposite was lower than that for PPy/TA that may be ascribed to the removal of CTAB from nanocomposite.

### 3.4 Surface area

Brunauer–Emmett–Teller (BET) measurements were performed to have a deeper understanding of the specific surface area of the composites and the N_2_ adsorption–desorption isotherms are shown in Fig. 5C. In all samples the nitrogen absorption were gradually increased from low pressure of about 0.009 to 0.8 (in case of PPy/TA/CTAB) or 0.9 (in case of PPy and PPy/TA), and then followed by a sharp rise due to the substantial interparticle porosity.^37^ Characteristic features of all samples showed type IV adsorption isotherms with a H3 type and very narrow hysteresis loop suggesting more uniformly and slit-shaped pores. However PPy/TA/CTAB displayed hysteresis loop at a lower value of pressure (*p*/*p*°) confirming smaller size of pores (mesopores) with almost regular geometry that is confirmed by data calculated from BET measurements (Table 2). The composite of PPy/TA/CTAB showed the largest surface area of 23.5 m^2^ g^−1^ compared to the bare PPy and composite of PPy/TA which were 8.2 m^2^ g^−1^ and 1.5 m^2^ g^−1^ respectively. Such difference corresponds to the variation of the pore size and volume. The adsorption is very low at 50 °C, the adsorption and desorption branches are close to zero adsorbate value.

**Table 2:** BET data of PPy, PPy/TA and PPy/TA/CTAB composites

Sample	*A*_BET_ [m^2^ g^−1^]	Total pore volume [cm^3^ g^−1^]	*Ø* pore width [nm]
PPy	8.17	0.50	246.66
PPy/TA	1.52	0.12	325.48
PPy/TA/CTAB	23.49	0.28	47.47

## 4. Conclusions

It has been shown that the physical and electrochemical properties of polypyrrole were enhanced in the presence of tannin and cationic surfactant of CTAB. The thermal stability enhancement of PPy was observed in the presence of TA. However, the addition of CTAB to the composite again decreased the thermal stability of the nancomposite. The modified electrode using PPy/TA/CTAB nanocomposite displayed a high surface area and porosity which resulted in higher current response and low resistant as confirmed by EIS data. Diffusion controlled process on the surface of the electrode was confirmed by studying scan rate on the modified electrode. All these data shows that this modified electrode is a good candidate for sensor applications. In addition, tannin acts as a dopant for PPy which later repels unwanted biomolecules such as ascorbic acid and could be used in selective biosensor applications.

## Conflicts of interest

There are no conflicts to declare.

## Supplementary Material

## References

[cit1] Khanbabaee K., van Ree T. (2001). Nat. Prod. Rep..

[cit2] Bharudin M. A., Zakaria S., Chia C. H. (2013). AIP Conf. Proc..

[cit3] Huang X. D., Liang J. B., Tan H. Y., Yahya R., Long R., Ho Y. W. (2011). J. Agric. Food Chem..

[cit4] Tiemann T. T., Lascano C. E., Kreuzer M., Hess H. D. (2008). J. Sci. Food Agric..

[cit5] Rhazi N., Hannache H., Oumam M., Sesbou A., Charrier B., Pizzi A., Charrier-El Bouhtoury F. (2015). Arabian J. Chem..

[cit6] Santos-Buelga C., Scalbert A. (2000). J. Sci. Food Agric..

[cit7] Bravo L. (1998). Nutr. Rev..

[cit8] Edeoga H. O., Okwu D. E., Mbaebie B. O. (2005). Afr. J. Biotechnol..

[cit9] Perumal Samy R., Gopalakrishnakone P. (2010). J. Evidence-Based Complementary Altern. Med..

[cit10] Avachat A., Avachat A. M., Dash R. R., Shrotriya S. N. (2011). Indian J. Pharm. Educ. Res..

[cit11] Saxena M., Saxena J., Nema R., Singh D., Gupta A. (2013). J. Pharmacogn. Phytochem..

[cit12] Hoong Y. B., Paridah M. T., Loh Y. F., Jalaluddin H., Chuah L. A. (2011). Int. J. Adhes. Adhes..

[cit13] Peres R. S., Cassel E., Azambuja D. S. (2012). ISRN Corros..

[cit14] Jiang L., Xie Q., Li Z., Li Y., Yao S. (2005). Sensors.

[cit15] Piovesan J. V., de Lima C. A., Santana E. R., Spinelli A. (2017). Sens. Actuators, B.

[cit16] Migahed M. D., Fahmy T., Ishra M., Barakat A. (2004). Polym. Test..

[cit17] Migdalski J., Błaż T., Lewenstam A. (2014). Electrochim. Acta.

[cit18] Upadhyay J., Kumar A., Gogoi B., Buragohain A. K. (2014). Synth. Met..

[cit19] Esmaeili C., Abdi M. M., Mathew A. P., Jonoobi M., Oksman K., Rezayi M. (2015). Sensors.

[cit20] Hoong Y. B., Paridah M. T., Luqman C. A., Koh M. P., Loh Y. F. (2009). Ind. Crops Prod..

[cit21] Zhang X., Zhang J., Song W., Lu Z. (2006). J. Phys. Chem. B.

[cit22] Wan M. , in Conducting Polymers with Micro or Nanometer Structure, 2008, pp. 88–157

[cit23] Yan X., Gu Y., Li C., Tang L., Zheng B., Li Y., Zhang Z., Yang M. (2016). Biosens. Bioelectron..

[cit24] Xie Q., Kuwabata S., Yoneyama H. (1997). J. Electroanal. Chem..

[cit25] Guo X., Kulkarni A., Doepke A., Halsall H. B., Iyer S., Heineman W. R. (2012). Anal. Chem..

[cit26] David T.-O., Pandiyan T., García-Ochoa E. M. (2007). Mater. Sci..

[cit27] Zhang X., Zhang J., Robinson C. (2004). Chem. Commun..

[cit28] Bhat N. V., Gadre a. P., Bambole V. a. (2001). J. Appl. Polym. Sci..

[cit29] Eisazadeh H. (2007). World J. Chem..

[cit30] Mao H., Liang J., Zhang H., Pei Q., Liu D., Wu S., Zhang Y., Song X. M. (2015). Biosens. Bioelectron..

[cit31] Naima R., Oumam M., Hannache H., Sesbou A., Charrier B., Pizzi A., El F. C. (2015). Ind. Crops Prod..

[cit32] Ping L., Pizzi A., Guo Z. D., Brosse N. (2012). Ind. Crops Prod..

[cit33] Lee W.-J., Lan W.-C. (2006). Bioresour. Technol..

[cit34] Nystrom G., Mihranyan A., Razaq A., Lindstrom T., Nyholm L., Strømme M. (2010). J. Phys. Chem. B.

[cit35] Danarto Y. C. , Prihananto S. A. and Pamungkas Z. A., in Prosiding Seminar Nasional Teknik Kimia ‘Kejuangan’, 2011, pp. 1–5

[cit36] Goworek J., Kierys A., Gac W., Borówka A., Kusak R. (2009). J. Therm. Anal. Calorim..

[cit37] Ramimoghadam D., Bin Hussein M. Z., Taufiq-Yap Y. H. (2012). Int. J. Mol. Sci..

